# Novel gradient echo sequence-based amide proton transfer magnetic resonance imaging in hyperacute cerebral infarction

**DOI:** 10.3892/mmr.2015.3165

**Published:** 2015-01-08

**Authors:** DEXIAO HUANG, SHENKAI LI, ZHUOZHI DAI, ZHIWEI SHEN, GEN YAN, RENHUA WU

**Affiliations:** Department of Medical Imaging, The Second Affiliated Hospital, Medical College of Shantou University, Shantou, Guangdong 515041, P.R. China

**Keywords:** hyperacute ischemia, amide proton transfer, chemical exchange saturation transfer, pH, stroke

## Abstract

In the progression of ischemia, pH is important and is essential in elucidating the association between metabolic disruption, lactate formation, acidosis and tissue damage. Chemical exchange-dependent saturation transfer (CEST) imaging can be used to detect tissue pH and, in particular, a specific form of CEST magnetic resonance imaging (MRI), termed amide proton transfer (APT) MRI, which is sensitive to pH and can detect ischemic lesions, even prior to diffusion abnormalities. The critical parameter governing the ability of CEST to detect pH is the sequence. In the present study, a novel strategy was used, based on the gradient echo sequence (GRE), which involved the insertion of a magnetization transfer pulse in each repetition time (TR) and minimizing the TR for *in vivo* APT imaging. The proposed GRE-APT MRI method was initially verified using a tissue-like pH phantom and optimized MRI parameters for APT imaging. In order to assess the range of acute cerebral infarction, rats (n=4) were subjected to middle cerebral artery occlusion (MCAO) and MRI scanning at 7 telsa (T). Hyperacute ischemic tissue damage was characterized using multiparametric imaging techniques, including diffusion, APT and T_2_-Weighted MRI. By using a magnetization transfer pulse and minimizing TR, GRE-APT provided high spatial resolution and a homogeneous signal, with clearly distinguished cerebral anatomy. The GRE-APT and diffusion MRI were significantly correlated with lactate content and the area of cerebral infarction in the APT and apparent diffusion coefficient (ADC) maps matched consistently during the hyperacute period. In addition, compared with the infarction area observed on the ADC MRI map, the APT map contained tissue, which had not yet been irreversibly damaged. Therefore, GRE-APT MRI waa able to detect ischemic lactic acidosis with sensitivity and spatiotemporal resolution, suggesting the potential use of pH MRI as a surrogate imaging marker of impaired tissue metabolism for the diagnosis and prognosis of hyperacute stroke.

## Introduction

Ischemic stroke is a severe disease, which often results in mortality or disability. Previous studies on ischemic stroke have demonstrated that thrombolytic treatment increased the survival rate and reduced disability in stroke patients (1). However, early intervention, which stimulates the reperfusion of ischemic penumbra is essential for effective therapeutic outcomes following ischemic stroke (1,2). Therefore, the availability of non-invasive imaging technologies, which are able to rapidly and specifically evaluate ischemic penumbra, are required for the progression of therapeutic research into acute stroke. The penumbra has been previously defined as a large area of metabolically-impaired perfusion deficit, which is able to retain cellular polarization (3,4). In the last 20 years, perfusion-weighted imaging (PWI) and diffusion-weighted imaging (DWI) in magnetic resonance imaging (MRI) have become the most well-established imaging techniques for detecting regions of reduced blood flow and cytotoxic edema, respectively (5,6). Furthermore, PWI/DWI mismatch is considered to represent ischemic tissue, which has not yet undergone severe tissue damage and has been used surgically to define the ischemic penumbra (7–9). However, ischemic tissue damage is complex and multifactorial and PWI/DWI mismatch provides only an approximation of the penumbra (10,11). In addition, the diagnostic accuracy of PWI/DWI mismatch remains controversial. Therefore, novel imaging techniques are required to augment existing penumbral imaging, provide greater insight into disease pathophysiology and assist in guiding treatment decisions.

Ischemic stroke induces a complex cascade of metabolic disruption in the cerebral tissue, which alters oxygen and glucose metabolism, tissue acidosis, adenosine triphosphate depletion and, ultimately, cerebral infarction (12–15). During ischemia, anaerobic metabolism and the formation of lactate lead to a decreased pH in the ischemic area. The disruption of metabolic processes may then be further exacerbated by hypoperfusion and the reduced buffering capacity of bicarbonate at an acidic pH, which may result in further tissue acidification (16,17). As energy metabolism is essential for cell viability and is disrupted at levels of blood flow that are higher than those that cause infarction, monitoring tissue metabolism may offer additional insights into early ischemia prior to irreversible damage (18).

As the chemical exchange between amide protons and bulk tissue water is pH-dependent, amide proton chemical exchange saturation transfer (CEST) MRI is able to assess tissue pH *in vivo* (19–23). In particular, a specific form of CEST MRI, termed amide proton transfer (APT) MRI, has been identified as sensitive to pH and can detect ischemic lesions prior to diffusion abnormalities (2,23–27). However, the sensitivity of CEST MRI requires enhanced due to the weak CEST effects (28–35). The sequence and optimization of parameters are the key features of the CEST experiments to focus on in the improvement of CEST sensitivity. The present study used a novel strategy based on the gradient echo sequence (GRE) combined with magnetic transfer and an *in vivo* rat acute cerebral infarction model was used to detect the ischemic penumbra using GRE-APT MRI.

## Materials and methods

### Phantom preparation

A multi-pH CEST gel phantom composed of creatine and low gelling point (LGP) agarose (Sigma-Aldrich, St. Louis, MO, USA) was prepared, as previously described (20). In brief, 1% agarose was added to phosphate-buffered saline (PBS; Oxoid, Basingstoke, UK). The phantom was microwave-heated to boiling and then cooled for ~25 mins in a water bath set at 50°C (Cole-Palmer, Vernon Hills, IL, USA). Creatine (Sigma-Aldrich) was then added to the gel solution to final concentrations of 50, 100 and 200 mM, followed by serial titration to pH 7.0 (EuTech Instruments, Ayer Raja, Singapore). The solutions were transferred into separate 10 ml centrifuge tubes for each creatinine concentration, which were then sealed and inserted into a phantom holder (63 mm Swift Cradle; Agilent Technologies, Inc., Santa Clara, CA, USA). A central tube filled with 1% agarose-gel in the absence of creatine was used as a background control.

### Acute stroke animal model

The study was approved by the ethics committee of the Second Affiliated Hospital of Shantou University Medical College, Guangdong, China. The protocol was approved by the Institutional Animal Care and Use Committee. Adult male Sprague-Dawley (SD) rats (n=4) aged 7–8 weeks (190–210 g) were obtained from Shantou University Medical College Laboratory Animal Center (Shantou, China) and kept in a 10/14 h light/dark cycle at 22–26°C with access to 30–40g rat diet and 60–70 ml water per day. The rats underwent imaging within 0.5–3 h, and then again 24 h following permanent filament middle cerebral artery occlusion (MCAO), while remaining under ~1.5% isoflurane-induced anesthesia (Shandong Keyuan Pharmaceutical Co., Ltd, Jinan, China), as previously described (2). The heart rate and blood oxygen partial pressure of the rats were monitored online using a real-time Nonin Pulse Oximeter 8600 (Nonin Medical, Inc., Plymouth, MN, USA). Standard permanent MCAO was induced by inserting a 4-0 nylon suture (Monosof 4-0; Tyco Healthcare, Zaltbommel, Netherlands) into the lumen of the internal carotid artery in order to block the origin of the middle cerebral artery. Following MRI using an Agilent 7.0 T animal MRI scanner (Agilent Technologies, Inc.) during the period of acute ischemia between 0.5 and 3 h, the animals were revived and provided with free access to food and water. The same anesthesia procedure was then repeated for a 24 h post MCAO follow-up MRI. T_2_-weighted hyperintensity, which has previously been confirmed as a reliable approach (33), was used to quantify the levels of infarction by comparing the abnormal MR signal from the MCAO side to that of the normal brain tissue.

### MRI parameters

Using an Agilent 7.0 T animal MRI scanner (Agilent Technologies, Inc.), the *in vitro* scanning parameters, including repetition time (TR) value, echo time (TE) and flip angle (FA) values of the gradient echo (GRE); FA value and duration time (T) of the presaturation pulse and the average readout direction × phase-encoding direction (ROxPE), were assessed to determine the optimal GRE parameter for hyperacute cerebral infarction. All experiments were performed using an Agilent 7T/160 animal scanner with a volume radiofrequency (RF) coil. The routine imaging parameters were set as follows: FA, 20°; TR, minimum 26.6 ms; TE, minimum 2.31 ms; slice thickness, 2 mm; field of view, 60×60 mm; number of averages, 1 and acquisition bandwidth, 50 kHz. A GRE sequence with magnetization transfer was used. The presaturation time was 20 ms and the FA was ~180°. The total scan duration changed according to the matrix (frequency coding number × phase coding number) which determines the resolution of the MR image. A duration of only 2.6 sec was required for imaging with a 64×64 mm matrix. All images were processed using the intrinsic VnmrJ 3.1A workstation (Agilent Technologies, Inc.) and Matlab, version 7.13 (Mathworks, Natick, MA, USA).

### Method for CEST imaging

CEST imaging was processed using asymmetric analysis. Three images were acquired, as follows: A normal T1 image without RF offset (I_0_); a T1 image with RF offset at the resonance of the labile protons (I_lib_; 3.5 ppm) and a T1 image with RF offset at the negative resonance point relative to water (I_ref_; −3.5 ppm). The CEST imaging (I_CEST_) was then calculated using the following formula (20):

$${\text{I}}_{\text{CEST}}=({\text{I}}_{\text{ref}}{\text{-I}}_{\text{lib}})/{\text{I}}_{0}$$

## Results

### Determination of the Z-spectrum

**Figure 1:**
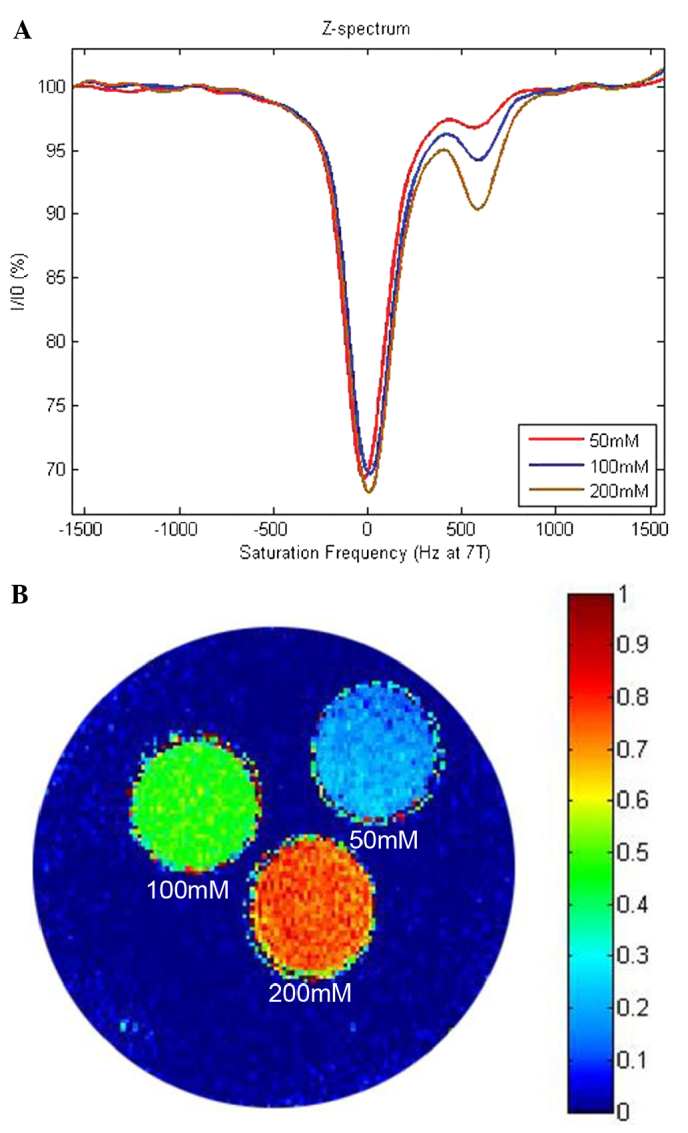
Z-spectrum obtained by monitoring a tissue-like pH phantom. (A) A marked CEST effect was identified at 1.87 ppm (516 Hz at 7.0 T). (B) CEST effect and signal intensity of phantom tubes were positively correlated with creatine concentration. CEST, chemical exchange-dependent saturation transfer.

**Figure 2:**
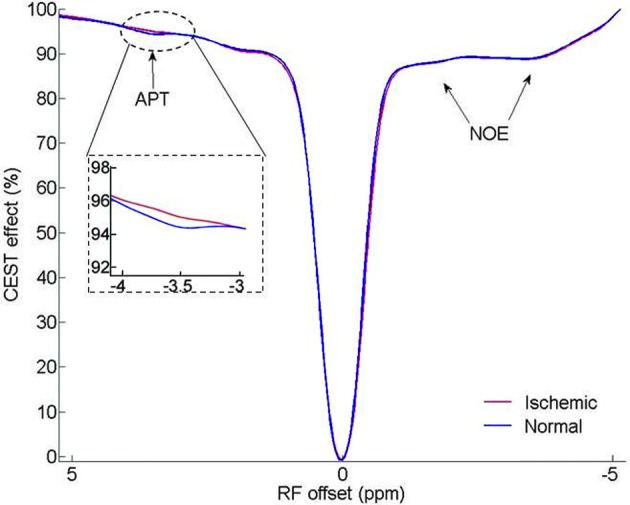
A Z-spectrum was obtained by monitoring the tissue water signals of Sprague-Dawley rats using 7.0 T magnetic resonance imaging *in vivo*. The maximal change in Z-spectral intensity was observed at ~3.5 ppm (1,050 Hz at 7.0 T), which represented a composite amide proton chemical shift. The NOE indicates the transfer of nuclear spin polarization from one nuclear spin population to another, via cross-relaxation. CEST, chemical exchange-dependent saturation transfer; RF, radiofrequency; APT, amide proton transfer; NOE, nuclear Overhauser enhancement effect.

The APT MRI method was initially verified using a tissue-like pH phantom to optimize the APT imaging parameters at 7.0 T MRI (36). The Z-spectrum, obtained through monitoring the tissue-like pH phantom, presented a clear CEST effect at ~1.87 ppm (516 Hz at 7.0 T) (Fig. 1A). In addition, the CEST effect and signal intensity of the phantom tubes was positively correlated with creatine concentration (Fig. 1B). The GRE-APT MRI measurements were markedly altered in global ischemia. As shown in Fig. 2, the Z-spectrum of the SD rats was obtained *in vivo* by detecting the tissue water signal at 7.0 T MRI, following optimization of the MRI parameters, including FA, TR, TE, slice thickness, field of view, number of excitations and acquisition bandwidth, for sensitizing the proton exchange of the endogenous proteins/peptides. The maximal change in Z-spectral intensity was observed at 3.5 ppm (1,050 Hz at 7.0 T), which was obtained through processing with a fat saturated frequency (Fsatfrq) function and corresponded to specific amide protons representing a composite amide proton chemical shift. This indicated that tissue pH can be assessed and imaged using GRE-APT MRI. A change in the Z-spectra was also observed a change at −3.5 ppm, where the CEST effect was 94.4% on the normal side of the cerebrum and increased to 95.6% on the ischemic side during the hyperacute period. This was consistent with the hypothesis that endogenous amide proton exchange occurred primarily due to changes in intracellular pH (Fig. 2).

### APT MRI of normal SD rats

**Figure 3:**
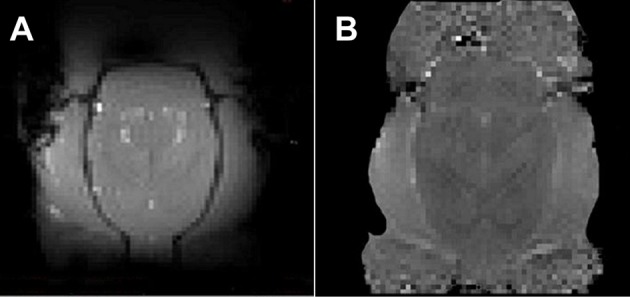
pH-weighted APT magnetic resonance imaging of a normal subject at 7.0 T. (A) MT image obtained with RF applied at −3.5 and 3.5 ppm (1,050 Hz at 7.0 T), and without RF irradiation. (B) The APT imaging was obtained with RF irradiation and via processing with Vnmr J 3.1A and Matlab software, which provide high spatial resolution and a homogeneous signal, and enable resolution of the cerebrum anatomy. APT, amide proton transfer; RF, radio frequency.

In a pilot APT MRI investigation of normal SD rats (n=3) *in vivo,* the APT imaging was calculated as the magnetization transfer ratio (MTR) asymmetry: I_ref_−I_libl_/I_0_, in which I_ref_ and I_lib_ denote the reference and labile protons, respectively, with RF applied at −3.5 and 3.5 ppm (1,050 Hz at 7.0 T). I_0_ denotes the scan without RF irradiation (Fig. 3). Fig. 3A is an MT image obtained with RF applied at −3.5 and 3.5 ppm (1,050 Hz at 7.0 T), and without RF irradiation. Fig. 3B shows the APT imaging obtained with RF irradiation and via processing with Vnmr J 3.1A and Matlab software. This method of imaging provided high spatial resolution and a homogeneous signal, which provided an appropriate resolution to distinguish the cerebral anatomy. The GRE-APT MRI appeared reasonably homogeneous within the cerebrum and may be applied for the detection of subtle pH lesions in hyperacute stroke.

### APT MRI of SD rats following the induction of MCAO

**Figure 4:**
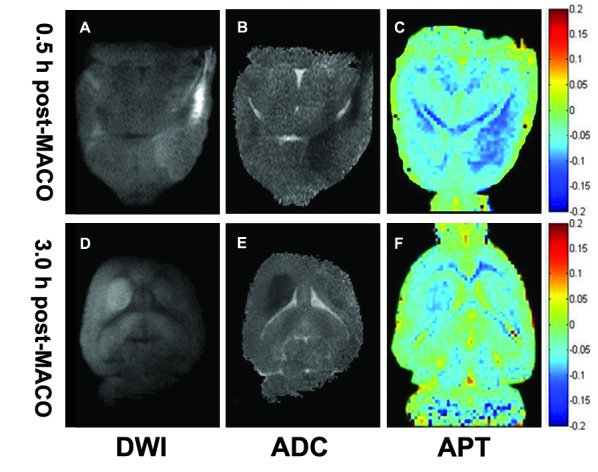
DWI and pH-weighted endogenous APT MRI of a representative hyperacute stroke (between 0.5 h and 3.0 h post-MCAO) animal. DWI, ADC and APT MRI images of rats (A–C) 0.5 h and (D–F) 3 h post-MCAO, respectively. Deficits of the APT and ADC maps matched consistently during the hyperacute period. Unlike the ADC map, the APT map exhibited an undefined deficit boundary and a gradient difference in lesion signal, with marked signal in the center and weak signal in the periphery during the hyperacute period. APT, amide proton transfer; MRI, magnetic resonance imaging; MCAO, middle cerebral artery occulsion; ADC, apparent diffusion coefficient; DWI, diffusion-weighted imaging.

All four MCAO rats observed in the present study demonstrated large deficits across the relatively hypoperfused region serviced by the right MCA, with corresponding deficits on the APT and apparent diffusion coefficient (ADC) maps (Fig. 4). However, no abnormal signals were observed on the T_1_ or T_2_-weighted images during the hyperacute period between 0.5 and 3.0 h. Furthermore, the deficits in the APT and ADC maps matched consistently during the hyperacute period, indicating severe energy breakdown and concomitant cell depolarization. All the hypoperfused regions progressed to complete infarction, which was confirmed by a 24 h-post-MCAO follow-up T_2_WI. Furthermore, the deficits on the APT and ADC maps were marginally increased during the hyperacute period. At 24 h-post-MCAO, the infarcted areas in the T2-weighted images corresponded with those of the hyperacute deficits on the APT maps. However, GRE-APT MRI may provide further insight into infarcted areas compared with ADC MRI. Unlike the ADC map, there were certain imaging characteristics with undefined deficit boundaries on the APT map and the gradient of hypointensity ranged, being marked in the center and weak in the peripheries during the hyperacute period. This observation may indicate that the infarction area on the ADC map contained tissue that had not undergone irreversible damage, as demonstrated in the imaging characteristics of the APT map.

## Discussion

CEST imaging, a novel molecular imaging technology, is able to non-invasively characterize the physical and physiological status of tissues *in vivo*, including tissue pH, APT, glycogen, glycosaminoglycan, lipid, enzymes and genes (28,37). The present study demonstrated that the sensitivity of CEST MRI was significantly improved by inserting a magnetization transfer pulse at each TR and by minimizing TR, based on the GRE sequence. As the effect of the pulses was cumulative, the CEST effect was enhanced and reached a steady state following multiple pulses. In addition, the present study verified this strategy using the tissue-like CEST phantoms at 7.0 T. In the Z-spectrum, the CEST effect was proportional to the concentration of the labile protons and the CEST effect increased with the phase-encoding number within a certain range, which suggested that this proposed novel method may enhance the effectiveness of pH-weighted APT MRI. Therefore, pH-weighted APT/CEST MRI based on a GRE sequence combined with magnetic transfer was capable of detecting ischemic lactic acidosis and provided APT imaging with sensitivity and spatiotemporal resolution, which was consistent with previous studies (21,26,38). In the present study, deficits were observed in the APT and ADC maps, which matched consistently and increased marginally during the hyperacute period. At 24 h-post-MCAO, the infarct area in the T2-weighted images corresponded with that of the APT map deficits observed between 0.5 and 3.0 h. In addition, compared with that of the conventional CEST imaging sequences (39–42), the method proposed in the present study demonstrated certain advantages, including a markedly reduced scan time, improving the temporal resolution of the APT imaging and enabling the imaging of patients with only minimal or no interference with their clinical care. In addition, the proposed method extended the interval of the saturation pulses, leading to a highly reduced specific absorption rate and enabling use of the interval to obtain a signal. Multislice imaging is also easily obtained. Overall, the method proposed by the present study may be easily translated in order to obtain CEST imaging on the majority of clinical MRI machines. The goal of future studies is to fully develop pH-weighted APT MRI and to evaluate its diagnostic use to ultimately augment the current diagnostic capability of hyperacute stroke.

The optimization of multiple MRI parameters is essential to improve the evaluation of ischemic tissue metabolism for predicting tissue outcomes and their response to potential treatments. Notably, pH-sensitive MRI may be beneficial in patients with tissues that are not irreversibly damaged. Previous animal studies have demonstrated that there may be a significant CBF/pH/ADC mismatch in acute ischemic stroke and that pH-weighted APT lesions may be used to improve the prediction of tissue outcomes (2). In addition, pH-weighted MRI may be able to delineate the PWI/DWI mismatch into a benign oligemic region and metabolic penumbra. However, the conventional CEST imaging sequence used for APT MRI *in vivo* provides only pH-weighted information. GRE-APT MRI using the modified sequence has been shown to provide additional insight into areas of infarction compared with ADC MRI and conventional CEST imaging sequences (39–42). The present study revealed APT map imaging characteristics with undefined deficit boundaries and a hypointensity gradient, which was more marked at the center and weaker at the peripheries during the hyperacute period. This indicated that the infarct area observed on the ADC map contained tissue which had not yet undergone irreversible damage. Within this pH-impaired region, it is likely that the metabolism was completely interrupted at the center and initially sustained at the peripheries. This may have been due to anaerobic glycolysis during the hyperacute period, leading to the accumulation of lactic acid and a reduction in local tissue pH, as indicated by the gradient of hypointensity in the APT MRI. The relatively weak signals in the lesion peripheries compared with those at the center may represent an area of impaired metabolism, which remained viable on the APT map, despite its identification as an infarct on the ADC map. These findings may be extended to the use of pH MRI as a surrogate imaging marker of impaired tissue metabolism and suggested that pH-weighted APT MRI may be used for the clinical diagnosis and determination of prognosis in patients with acute stroke.

The regulation of pH is important in the progression of disease, therefore, it is essential to elucidate the association between metabolic disruption, lactate content, acidosis and tissue damage. Various techniques have been developed for pH-weighted APT imaging *in vivo*, however, improvements are to provide higher sensitivity, signal-to-noise ratio and concentration independence (43). A pH-weighted APT MRI offers a more adaptable approach as exchange sites may be built into the agent molecules to make them concentration independent (44). In the present study, APT MRI was used to identify and characterize lesions in a permanent stroke model to examine the association between tissue pH, diffusion and lactic acidosis in acute shock. However, APT MRI may be extended to other diseases, including solid tumors and disorders of glycogen, glycosaminoglycan, lipid, enzymes and genes.

In conclusion, the present study characterized heterogeneous ischemic tissue damage using multiparametric MRI of pH, diffusion and relaxation imaging during the hyperacute period. A novel method was used, based on the GRE sequence combined with magnetic transfer and phase encoding, which demonstrated that pH-weighted APT provided high spatial resolution, a homogeneous signal and clearly distinguished cerebral anatomy. In addition, the pH-weighted APT and diffusion MRI were significantly correlated with lactate content and the deficits of the APT and ADC maps matched consistently during the hyperacute period. The GRE-APT MRI provided further insight into the infarction area compared with the ADC MRI, as reflected in certain lesions on the APT map exhibiting undefined deficit boundaries and a hypointensity gradient, which was more marked at the center and weaker at the peripheries during the hyperacute period. This indicated the presence of tissue within the penumbra, which had not yet undergone irreversible damage. Furthermore, the present study demonstrated that, due to its high sensitivity to tissue acidification, pH-weighted APT MRI using the gradient echo sequence provided surrogate imaging markers of impaired tissue metabolism. This suggested that pH-weighted APT MRI may be used for clinical diagnosis and for determining prognosis in patients with hyperacute stroke.
